# Lipoplatin Formulation Review Article

**DOI:** 10.1155/2012/581363

**Published:** 2011-08-29

**Authors:** G. P. Stathopoulos, T. Boulikas

**Affiliations:** ^1^First Oncology Clinic, Errikos Dunant Hospital, Athens, Greece; ^2^Regulon Inc. and Regulon AE, Afxentiou 7, Alimos, 17455 Athens, Greece

## Abstract

Patented platform technologies have been used for the liposomal encapsulation of cisplatin (Lipoplatin) into tumor-targeted 110 nm (in diameter) nanoparticles. The molecular mechanisms, preclinical and clinical data concerning lipoplatin, are reviewed here. Lipoplatin has been successfully administered in three randomized Phase II and III clinical trials. The clinical data mainly include non-small-cell lung cancer but also pancreatic, breast, and head and neck cancers. It is anticipated that lipoplatin will replace cisplatin as well as increase its potential applications. For the first time, a platinum drug has shown superiority to cisplatin, at least in non-squamous non-small-cell lung cancer as reported in a Phase III study which documented a simultaneous lowering of all of the side effects of cisplatin.

## 1. Introduction

Over thelast twenty years, the effort to produce new, more effective, and less toxic cytotoxic agents has been intensive, in order to ameliorate the treatment of cancer patients. One of the most effective agents since the late 1970s has been cisplatin (CDDP) in patients with testicular cancer [1], ovarian cancer [2], head and neck [3], and lung cancer [4] as well as bladder cancer [5] and in other malignancies [6, 7]. Cisplatin was established as being quite effective and as one of the most important cytotoxic agents. It has mainly been administered in combination with other agents. The toxicity rendered by cisplatin has been its main drawback, particularly nephrotoxicity [8–10]. After 1990, new agents that did not cause nephrotoxicity were produced as a substitute for cisplatin. Agents such as carboplatin [11] paclitaxel, docetaxel, gemcitabine, vinorelbine, and irinotecan [12–15] were used either in combination or as substitutes for cisplatin [16, 17]. These agents succeeded in producing no nephrotoxicity but did produce other toxicities such as myelotoxicity, in comparison to cisplatin. The main example was carboplatin, an analogue of cisplatin, which showed no renal toxicity but produced higher myelotoxicity than cisplatin. Carboplatin has often been used as a substitute for CDDP [11, 12] in lung [15], head and neck, and ovarian cancers [11]. The effectiveness of carboplatin was more or less equal to that of CDDP but not better. For instance, CDDP was shown to be more effective than carboplatin in the most common lung cancer, adenocarcinoma [18]. The other agents, previously mentioned, are mainly administered in combination with CDDP than as a substitute for it. Over all of the last twenty years, cisplatin has been in regular usage since most oncologists still believe it has priority with regard to effectiveness. Liposomal agents comprise another direction which research is taking and several of these have become part of clinical practice as is the case of liposomal anthracycline. None of these agents has managed to become a substitute for cisplatin, and they are used as second-line treatment. 

Our review article is related to a new formulation of cisplatin, that is, liposomal cisplatin (lipoplatin). The purpose of this agent is to become a substitute for the original cisplatin, and, thus, the two drugs must be compared with regard to toxicity and effectiveness.

There are preclinical data in cancer cell cultures and in animals as well as clinical data which involve Phase I studies, pharmacokinetics and Phase II and Phase III studies. The data in 16 published studies are related to patients with pancreatic cancer, non-small-cell lung cancer (NSCLC), head and neck, and breast cancers. 

## 2. Lipoplatin: Formulation, Mechanisms, and Technology

**Figure 1:**
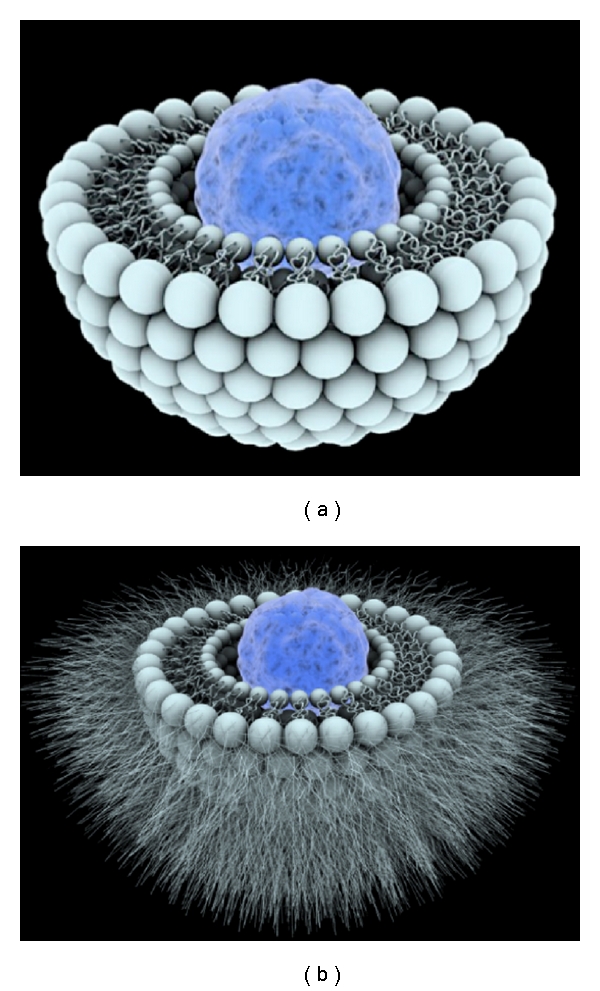
Depiction of a lipoplatin nanoparticle (b). Cisplatin molecules are depicted as blue spheres surrounded by the lipid bilayer with the PEGylated lipid sticking out like hair from the outer surface. Thus, this toxic substance, cisplatin, is camouflaged by its lipid shell as a nutrient. This nanoparticle can pass undetected by macrophages after intravenous injection to human cancer patients because of its PEG coating thus escaping immune surveillance [20]. © CNRS Photothèque/SAGASCIENCE/CAILLAUD François.

Cisplatin was formulated into liposomes as depicted in Figure 1. The lipids of lipoplatin are composed of soy phosphatidyl choline (SPC-3), cholesterol, dipalmitoyl phosphatidyl glycerol (DPPG), and methoxy-polyethylene glycol-distearoyl phosphatidylethanolamine (mPEG 2000-DSPE). The formulation was achieved by the formation of reverse micelles between cisplatin and DPPG under special conditions of pH, ethanol, ionic strength, and other parameters, and the cisplatin-DPPG reverse micelles were subsequently converted into liposomes by interaction with neutral lipids. About 15 extrusions are performed to give to the nanoparticles their final size of 110 nm, using a thermobarrel, extruder and membranes of 0.2, 0.1, 0.08 and 0.05 *μ*m pore sizes under ultra pure nitrogen pressure.

The nanoparticles, 110 nm in diameter, have the ability to target tumors and metastasis following intravenous administration using the compromised endothelium of the tumor vasculature sprouted during neoangiogenesis; this process, known as extravasation, takes advantage of the compromised endothelium of the vasculature of the tumors generated during neoangiogenesis. Lipoplatin has shown an amazing concentration in tumors and metastases at levels up to 200-fold higher compared to the adjacent normal tissue in surgical specimens from patients [19].

## 3. Molecular Mechanisms of Cisplatin and Lipoplatin

**Figure 2:**
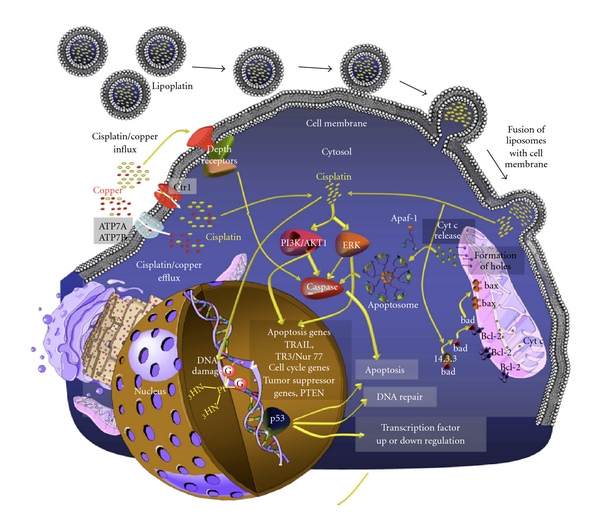
Penetration of lipoplatin nanoparticles through the cell membrane of tumor cells. lipoplatin nanoparticles once inside the tumor cell mass can fuse with the cell membrane because of the presence of the fusogenic lipid DPPG in their lipid bilayer; an alternative mechanism proposed is that lipoplatin is taken by *endocytosis* by tumor cells as shown from lipoplatin containing fluorescent lipids and imaging of the tumor cells in culture thus treated with fluorescent microscopy (see Figure 3). These processes occurring at the cell membrane level are promoted by the lipid shell of the nanoparticles (disguised as nutrients) [20].

After infusion, cisplatin is rapidly excreted in the urine causing renal tubular damage. When it reaches normal and malignant cells, it uses the major copper influx transporter Ctr1 for entry inside the cytoplasm (Figure 2). Ctr1 has been convincingly demonstrated to transport cisplatin and its analogues, carboplatin, and oxaliplatin. Two copper efflux transporters, ATP7A and ATP7B, regulate the efflux of cisplatin [21]. 

The S-containing tripeptide glutathione is present in cells at mM concentrations, and the formation of complexes with cisplatin plays an important role in its detoxification and biological activities. The depletion of glutathione levels has been shown to increase the toxicity of cisplatin to kidney cells. Cancer cells that are resistant to cisplatin often have elevated glutathione levels. Glutathione could quench DNA-Pt monofunctional adducts before they can rearrange toxic bifunctional adducts on DNA. Human glutathione S-transferase P1 (GSTP1) contributes to chemoresistance and its suppression, decreasing the cisplatin-induced activation of ERK1/2 and might have synergistic therapeutic effects [22].

Cisplatin and other apoptotic stimuli trigger the release of cytochrome c from the mitochondrial intermembrane space to the cytosol, which induces the formation of the apoptosome and the activation of procaspase-9, leading to apoptosis. The apoptosome is an Apaf-1 cytochrome c complex that activates procaspase-9. Cisplatin can also activate the proapoptotic protein Bax, resulting in cytochrome c release, caspase activation, and apoptosis; Bax activation is implicated in the nephrotoxicity of cisplatin [23]. Bcl-2 plays an important role in the mitochondrial apoptotic pathway. Although the general role of Bcl-2 is antiapoptotic, Bcl-2 fragments resulting by caspase cleavage after cisplatin treatment of cells in culture could promote the apoptotic process [24]. Lipoplatin, releasing cisplatin molecules in the cytoplasm of the tumor cell, is also proposed to activate the mitochondrial apoptotic cascade.

During signal transduction, a cell senses both the external and internal environment and converts a stimulus into an ordered sequence of phosphorylation-dephosphorylation, protease degradation, gene regulation, or ion flux events, across the cell membrane. Receptor tyrosine kinases contribute to chemoresistance in tumors. A number of additional properties of cisplatin are now emerging including the activation of signal transduction pathways leading to apoptosis. The firing of such pathways may originate at the level of the cell membrane after damage of the receptor or lipid molecules by cisplatin, in the cytoplasm by modulation of proteins via the interaction of their thiol groups with cisplatin, (kinases, and other regulatory proteins and enzymes), or finally from DNA damage via the activation of the DNA repair pathways [25, 26]. 

Cisplatin induction of signaling is cell type, time and dose dependent. It induces oxidative stress and is an activator of stress-signaling pathways especially of the mitogen-activated protein (MAP) kinase cascades. The extracellular signal-regulated kinase (ERK) pathway is indeed activated by cisplatin. The acquisition of cisplatin resistance by ovarian carcinoma cells was associated with the loss of ERK activation in response to cisplatin [27]. ERK activation and DNA-damage induced apoptosis are tightly linked; p53 may act as one of the upstream regulators of ERK activation for the induction of apoptosis in carboplatin-treated cervical cancer cells [28]. The treatment of cells with high cisplatin concentrations (one order of magnitude higher than the IC_50_) induces cellular superoxide formation and caspase activation independently of nuclear DNA damage. In contrast, cisplatin concentrations at IC_50_ doses, which do not induce acute apoptosis, are sufficient for the induction of DNA damage signaling [29].

 The PI3K/Akt cascade has an important role in the resistance of ovarian cancer cells to cisplatin, and the inhibition of PI3K/Akt increases the efficacy of cisplatin [30]. The Akt-specific inhibitor LY294005 increased the efficacy of docetaxel, did not affect the efficacy of 6-thioguanine, and decreased the efficacy of cisplatin, lipoplatin, oxaliplatin, and lipoxal in human colorectal adenocarcinoma sublines, suggesting a novel property of Akt in aggravating drug sensitivity [31].

Cisplatin appears to exhibit synergistic effects with other potent inducers of apoptosis such as a synthetic isothiocyanate; the sequential administration of both agents led to increased intracellular platinum accumulation, glutathione depletion, poly (ADP-ribosyl) polymerase cleavage, stimulation of caspase-3 activity, upregulation of p53, FasL and Gadd45alpha, cyclin B1 downregulation, and an increase in mitogen-activated protein kinases JNK, ERK, and p38 phosphorylation as well as PI3K level alterations [32].

## 4. Resistance of Tumor Cells to Cisplatin and a Role for Lipoplatin

The resistance of tumor cells to cisplatin is attributed to at least four different mechanisms: (i) decreased levels of cisplatin entrance to the cytoplasm or increased efflux through the cell membrane, (ii) increased levels of glutathione, (iii) modulation of signaling pathways, and (iv) enhanced levels of DNA repair. 

However, additional pathways have been found for establishing the cisplatin resistant phenotype. For example, the selection of ovarian carcinoma cells in culture in the presence of cisplatin led to upregulated expression of the L1 adhesion molecule; this could constitute a mechanism for the establishment of chemoresistance and of a more malignant tumor phenotype [33]. 

The direct fusion of lipoplatin nanoparticles with the membrane of the tumor cell (Figure 2) suggests that lipoplatin can have applications after the failure of cisplatin front-line chemotherapy and the development of cisplatin resistance at the cell membrane level.

## 5. Preclinical Studies

A comparison of the cytotoxicity of lipoplatin and cisplatin in vitro in established cell lines (derived from NSCLC, renal cell carcinoma, and in normal hematopoietic cell precursors), as well as the identification of biological markers associated with sensitivity and resistance has rendered some interesting data. ERCC1 and LRP expression levels appeared to be valid predictors of sensitivity or resistance to both drugs. A superior cytotoxicity in all tumor cell models and a much lower toxicity in normal cells for lipoplatin compared with cisplatin were found, suggesting a higher therapeutic index for the liposomal compound [34].

Fedier et al. [35] investigated whether the cytotoxic effect of lipoplatin is dependent on the functional integrity of DNA mismatch repair (MMR). MMR is a postreplicative DNA repair mechanism implicated in cell cycle control and apoptosis. MMR function was found to be a relevant determinant accounting for the cytotoxicity of lipoplatin [35]. A possible relationship between MMR-mediated cisplatin DNA damage signaling, and the Akt signaling pathway was also found [31]. 

**Figure 3:**
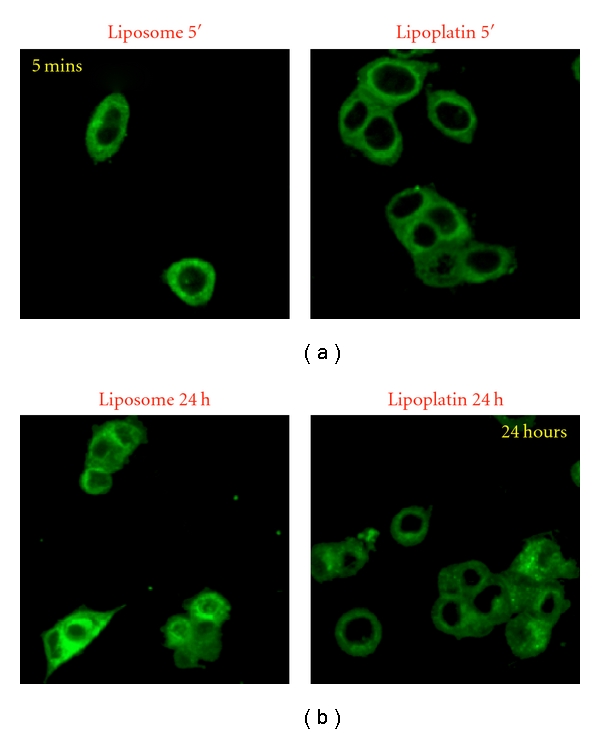
Lipoplatin or DPPG-liposomes with fluorescent lipids enter rapidly MCF-7 breast cancer cells in culture. Time-course processing of FITC-labeled DPPG-containing liposomes (a) and Lipoplatin (b) using confocal microscopy. At 5 min, the majority of the signal is localized in the membrane. Lipids are rapidly internalized and at 4–24 hours, a strong signal is observed in the cytoplasm and at the perinuclear area. These results demonstrate that *lipoplatin or DPPG-liposomes* nanoparticles are able to cross the cell membrane barrier [20].

The fusion between liposomes and the cell membrane was suggested based on the fusogenic properties of DPPG and lipids integrated into the shell of lipoplatin (Figure 2). Subsequent cell culture studies where the lipids of the lipoplatin nanoparticle were labelled with fluorescein isothiocyanate (FITC) established the rapid uptake and internalization of the nanoparticles (Figure 3). In these studies the fluorescent nanoparticles were incubated with MCF-7 breast cancer cells in culture for various times ranging from 5 min to 24 h, and the cells were fixed and visualized by confocal microscopy (Figure 3). Liposomes containing DPPG without cisplatin were also used as a control. The study has provided proof that the lipids of lipoplatin labelled with FITC are transferred initially (in less than 5 min) to the cell membrane of MCF-7 cells in culture and are then (from 5 min to 24 h) docked to the interior of the cell. The membrane fusion is proposed to modulate signalling, an important process for cancer cell proliferation.

The lower nephrotoxicity of lipoplatin, compared to cisplatin, was shown in mice, rats, and SCID mice [36], whereas animals injected with cisplatin developed renal insufficiency with clear evidence of tubular damage, but those injected with the same dose of lipoplatin were almost completely free of kidney injury [36]. 

**Figure 4:**
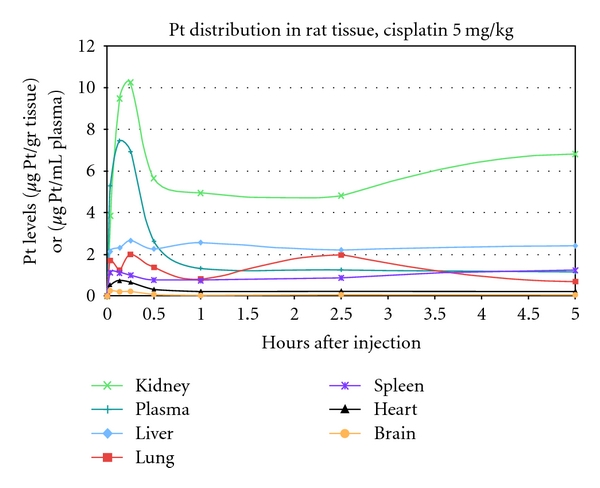
Kidney and other tissue accumulation of total platinum after *cisplatin* injection of rats (0–5h) [20].

In order to explain the lower toxicity of lipoplatin compared to cisplatin, the levels of total platinum in rat tissue after cisplatin or lipoplatin injections were determined at different time intervals. The maximum levels of total platinum after cisplatin were found in the kidney followed by the plasma, liver, lung, spleen, heart, and brain, in those tissues examined from  5 min to 5 h. At later times (up to 50 h), the order of the tissues with the highest levels of platinum was the kidney, liver spleen, plasma, lung, heart, and brain. A single treatment with 30 mg/kg lipoplatin in rats resulted in no toxicity, whereas 2 or 3 weekly administrations at 30 mg/kg in rats produced neutropenia but no nephrotoxicity. However, a single injection of 5 mg/kg cisplatin in rats resulted in severe nephrotoxicity. The levels of total platinum attained in animal kidneys after cisplatin administration are about the same as those after lipoplatin (Figure 4); however, at about 1 h and up to 5 days, the levels of total platinum are about 1 microgram after lipoplatin compared to 5 micrograms after cisplatin administration (Figure 4).

After cisplatin injection, the kidneys accumulate the highest levels of platinum among all of the animal tissues, followed by the liver and the lung. One hour after lipoplatin administration i.p., the kidney Pt levels drop from 13 to 3 *μ*g/g tissue. The highest Pt levels among all of the animal tissues are in the liver and spleen after 4 h i.p. administration maintained for over 100 h.

The treatment of dogs with lipoplatin led to the conclusion that the drug can be safely administered to clinically normal dogs at dosages of up to 150 mg/m^2^ without the need for concurrent hydration protocols. The maximum tolerated dose (MTD) of unencapsulated cisplatin in dogs has been established as 70 mg/m^2^. Therefore, lipoplatin would allow the safe and repeated administration of doses higher than the MTD of unencapsulated cisplatin [37].

The intrapleural administration of lipoplatin in an animal model seems to offer a more effective therapeutic index while improving tolerability. Wistar rats were treated with doses of 10 mg/kg lipoplatin (intravenously) versus 10 or 20 mg/kg lipoplatin (intrapleurally) corresponding to 60 and 120 mg/m^2^, respectively, in humans. The authors noted minor fibrotic changes in the pleura of rats injected intrapleurally, and mild kidney changes in rats injected intravenously, as expected [38].

## 6. Cellular Uptake and Cytoplasm/DNA Distribution of Cisplatin versus Lipoplatin

The antineoplastic or radio-sensitizing activity of platinum drugs is attributed to their binding to DNA. The time course of accumulation of cisplatin, lipoplatin, oxaliplatin, and lipoxal (liposomal oxaliplatin) in the human colorectal cancer HCT116 cell line and their distribution between the cytoplasm and DNA were measured by inductively coupled plasma mass spectrometry. The distribution of cytoplasm/DNA of free cisplatin and lipoplatin were similar. However, lipoxal displayed a higher accumulation in the cytoplasm compared to free oxaliplatin, consistent with its proposed mechanism of fusion with the cell membrane [39].

The cytotoxicity and synergic effect of platinum compounds with radiation were examined in F98 glioma cells. Lipoplatin improved the cell uptake of cisplatin by 3-fold, and its radiosensitizing potential was enhanced by 14-fold. Among the five platinum compounds tested, carboplatin and lipoplatin showed the best radiosensitizing effect. Lipoplatin seemed the most promising since it led to the best cellular incorporation and reduced all the toxicities of cisplatin [40].

## 7. Clinical Studies

### 7.1. Pharmacokinetics

In the administration of liposomal cisplatin to humans, the target was to determine the pharmacokinetics and adverse reactions. A Phase I study of 27 patients with different malignancies was performed. The drug was infused for 8 hrs every fourteen days at escalating doses. The drug levels started at 25 mg/m^2^ and were increased by 25 mg/m^2^ up to 125 mg/m^2^. Three-5 patients were selected for each dosage. Blood was taken at certain time intervals in order to estimate the total platinum plasma levels. For pharmacokinetics, blood was drawn at 0, 3, 6, 8, 12, 24 hrs and 3, 5, 7 days, into tubes containing EDTA, and total platinum levels (i.e., free plus proteins bound plus liposomal) were analyzed by atomic absorption. Total platinum was also determined in the ultrafiltrate of less plasma. The maximum level attained in the plasma was 5.7 *μ*g/mL at 8 hrs. The levels of platinum in the blood after lipoplatin infusion drop to normal on the fourth day at a dose of 100 mg/m^2^, but at a dose of 125 mg/m^2^ platinum can be detected in the blood for 7 days (Table 1) [41]. Renal function tests (blood urea, serum creatinine, and creatinine clearance) showed no change before and after treatment. The excretion of platinum in the urine in lipoplatin-treated patients attains a maximum within 8 hrs (infusion period) and declines thereafter. During the 3 following days (after infusion) 40.7% of the total platinum was excreted in the urine. Toxicity was very mild (grade 1 or 2 neutropenia and nausea/vomiting) at the 125 mg/m^2^ dosage level. In another trial, the tumor uptake of lipoplatin was examined in comparison to normal tissue, in 4 patients with hepatocellular adenocarcinoma, gastric cancer, and colon cancer. Lipoplatin was administered to the patients 24 hours before the surgery [19]. This study showed liposomal cisplatin accumulation in tumors as compared to normal tissue after the intravenous infusion of lipoplatin. Among the various surgical specimens examined, gastric tumors revealed the highest levels of total platinum (up to 262 *μ*g cisplatin/gr tissue). The liver metastatic specimen displayed a total amount of 131.15 *μ*g platinum/gr of tissue compared to 20.94 *μ*g platinum/gr of normal liver tissue. Both specimens of gastric tumors appeared to accumulate the highest amounts of platinum among all specimens analyzed in this study: 262.62 and 66.38 *μ*g/gr of tissue. The total platinum levels in the colon tumor specimens were 11.26 and 7.69 *μ*g platinum/gr of tissue compared to 0.06 *μ*g/gr normal colon tissue [19].

### 7.2. Dose-Limited Toxicity and Maximum Tolerated Doses

The human testing of this new agent primarily required the definition of toxicity by investigating the MTD as well as the dose-limited toxicity (DLT). Two Phase I and I-II studies examined these objectives. The first trial was in patients with advanced pancreatic cancer. The results showed that the dosages which began to produce side effects were 100 mg/m^2^ and 125 mg/m^2^. But these dosages did not later prove that this was the DLT since lipoplatin was combined with gemcitabine, the latter which may have been responsible for the toxicity [42]. The second study defined similar doses as the DLT and MTD. This trial also used two agents, lipoplatin and gemcitabine in pretreated patients with NSCLC. The two drugs were repeated on day 8. The small number of 13 patients was not efficacious enough to determine ample data concerning toxicity [43]. In both these aforementioned studies, there was also a defect in that all of the patients had undergone chemotherapy pretreatment when they were recruited and the efficacy of lipoplatin was tested. A proper third Phase I trial was eventually performed. The main objective of this study was to determine the DLT and MTD of lipoplatin tested as a single agent and in combination with a second cytotoxic agent. The selected second agent was paclitaxel. All of the patients had NSCLC. Adverse reactions, mainly myelotoxicity, renal toxicity and gastrointestinal toxicity (nausea, vomiting, diarrhea) were determined. Sixty-six patients were recruited and evaluated. Thirty-nine patients comprised the group that received lipoplatin monotherapy, and 27 patients were given lipoplatin in combination with paclitaxel. In the first group, the dosage of lipoplatin started at the level of 125 mg/m^2^ and the drug-dose escalation increased to 350 mg/m^2^. It was determined that 350 mg/m^2^ was the DLT and 300 mg/m^2^ the MDT. In the group that received combination therapy, the escalation of paclitaxel started at 100 mg/m^2^ and went up to 175 mg/m^2^ and of lipoplatin from 100 mg/m^2^ to 250 mg/m^2^. The results of the combined treatment evaluation determined the DLT as 250 mg/m^2^ and the MTD, 200 mg/m^2^. Nausea, vomiting, fatigue, and neutropenia were not higher than grade 1-2, and other adverse reactions in a small percentage of patients reached grade 3. In the combined modality, other side effects, such as neurotoxicity, were observed, and this was attributed to paclitaxel. Grade 1 nephrotoxicity was observed in a small percentage of patients, but this was only temporary (Table 2) [44].

Over the last five years, several Phase II and III trials have been performed in different institutions and countries. Lipoplatin has been tested in the following malignancies: pancreatic cancer, head and neck cancer, mesothelioma, breast and gastric cancer, and NSCLC. In pancreatic cancer, lipoplatin was administered as second-line treatment in combination with gemcitabine. The patients had initially undergone gemcitabine monotherapy as first-line treatment and were experiencing disease progression. The combination of lipoplatin with gemcitabine rendered a response rate of 8% [42].

A trial was done concerning a combination of lipoplatin 120 mg/m^2^ plus 5-fluorouracil 400 mg/m^2^ and leucovorin, both cytotoxic drugs administered weekly along with radiotherapy. The cytotoxic agents were given on day 1 and radiotherapy (dosage 3.5 Gy × 3, days 2, 3, 4) for four or five weeks. This treatment was given to patients with advanced gastric cancer. No serious toxicity was observed, and the therapy was well tolerated; 18.2% patients developed grade 1 renal toxicity and nausea and 25% showed fatigue. A good response to the combined treatment was observed [45].

It is too early to confirm that lipoplatin is effective in mesothelioma. There is a case report indicating the responsiveness of mesothelioma to lipoplatin given in combination with gemcitabine as second-line treatment on disease recurrence [46]. 

The testing of the toxicity and effectiveness of liposomal cisplatin was done in patients with squamous cell carcinoma of the head and neck. This was a randomized study comparing lipoplatin combined with 5-fluorouracil versus cisplatin combined with 5-fluorouracil. The toxicity was well tolerated. Grade 3 renal toxicity was much lower after lipoplatin administration than after cisplatin. Higher myelotoxicity was observed in the cisplatin arm (31.7% versus 12% in the lipoplatin arm). Mucositis and peripheral neuropathy were also much higher in the cisplatin group. The response rate was higher in the cisplatin arm, but stable disease was higher in the lipoplatin arm. This low responsiveness of the lipoplatin arm may be due to the quite low dosage administered and its short duration. One should take into account that the MTD is 200 mg/m^2^ and not 100 mg/m^2^ [47].

A Phase II trial combining lipoplatin with vinorelbine in first-line treatment of HER2/neu-negative metastatic breast cancer was done. The investigators administered the above agents on the basis of the rationale that the frequent use of anthracyclines and taxanes in the adjuvant setting of breast cancer has led to drug resistance and cardiac toxicity. This raised the need for new agents in the metastatic setting. Another reason for testing the aforementioned combination was that the use of cisplatin-vinorelbine showed interesting results with an overall response rate of 64%. The administered dose of lipoplatin was 120 mg/m^2^ and of vinorelbine 30 mg/m^2^. The objective response rate of the latter combination was 50% (one complete response). Stable disease was 45.5%. Toxicity was well tolerated [48].

One Phase II and two Phase III trials have been recently integrated and published. In these studies, lipoplatin was combined with a second agent in comparison with cisplatin also combined with the same second agent, and the objectives were to determine the side effects and efficacy. In the Phase II randomized study, lipoplatin (dosage 120 mg/m^2^ given on days 1, 8, 15) combined with gemcitabine (1000 mg/m^2^ given on days 1, 8) was compared with cisplatin (100 mg/m^2^ day 1) combined with gemcitabine (1000 mg/m^2^ given on days 1, 8). With respect to efficacy, the overall response rate of the lipoplatin arm was 31.7%, and the cisplatin arm 25.6%. Although the efficacy of lipoplatin was not statistically higher than that of cisplatin, a better response rate was achieved with lipoplatin, particularly in cases of adenocarcinoma. The more important finding was the toxicity outcome which was shown to be much lower in patients treated with lipoplatin versus in patients treated with cisplatin. Very low nephrotoxicity was observed in the patients who received lipoplatin. Although the aforementioned study [49] included a rather limited number of patients (88 in total), the results were confirmed by another study which was done in parallel to the above trial. 

These results with respect to the study done in parallel, mentioned above, are as follows: this Phase III trial included 229 evaluable patients. The differences between this study and the previous one were, the number of patients, the dosage of the drugs, the repetition of the courses, and the second agent which was combined with lipoplatin and cisplatin. The dose of lipoplatin was 200 mg/m^2^, which is the proper MTD combined with paclitaxel 135 mg/m^2^ repeated every 2 weeks for a planned 9 courses. The control arm received cisplatin 75 mg/m^2^ also combined with paclitaxel 135 mg/m^2^, repeated every 2 weeks. The planned number of courses was 9. The treatment of both agents and arms was on day 1. The main objectives of this trial were to determine the toxicity and median survival. The results were quite impressive; nephrotoxicity, in particular, leukopenia, nausea/vomiting, and asthenia were statistically significantly lower after lipoplatin treatment (*P* ≤ 0.001, 0.017, 0.042, 0.019, resp.) (Table 3). The comparison of efficacy was also important; the response rate was 59.7% for the lipoplatin arm, and 47% for the cisplatin arm (no statistically significant difference, *P*  =  0.073) (Table 4). The median and overall survival for both arms was the same [50].

The data documented in the last two trials indicate that the cisplatin formulation (lipoplatin) could be considered as the best substitute for cisplatin, at least in NSCLC, with regard to efficacy and toxicity. 

The next Phase III trial was based on certain indications from the previous trials, and this was the possibility that NSCLC subtypes may have a different response rate with the administration of lipoplatin or cisplatin. This study recruited patients with nonsquamous cell lung cancer, mainly adenocarcinomas, and they were treated with lipoplatin combined with paclitaxel versus cisplatin combined with paclitaxel. The dosage and administration of these two combined treatments was the same as in the previous study. It was found in 202 patients randomized into two groups, that the response rate was superior in the lipoplatin group. The difference was statistically significant (*P* = 0.036) (Table 5). The median survival for the lipoplatin group was 10 months and for cisplatin group 8 months, approaching statistical significance (*P* = 0.1551) [51].

There are data examining the possibility of using lipoplatin in cancer patients with renal failure. The preliminary data show that patients with serum creatinine ranging from 1.6–3.5 mg/dL tolerate lipoplatin without increasing renal failure and without side effects such as neutropenia, nausea/vomiting, and fatigue.

## 8. Conclusion

The efforts over the last 20 years to produce a substitute for cisplatin, a very important and effective anticancer agent, with a similarly effective and less toxic agent, have not properly succeeded. The current data in a number of preclinical and clinical trials shed new light on the previous efforts to produce a substitute for cisplatin. Liposomal cisplatin (lipoplatin), is a new formulation of cisplatin and one would expect at least to achieve equal effectiveness. Phase I, II, and III trials have shown lipoplatin to produce similar efficacy to that of cisplatin in pancreatic, head and neck, breast cancers, and NSCLC (the latter has been more broadly tested). In a new substitute for cisplatin, what is more important, apart from effectiveness, is significant toxicity reduction. The reduction of toxicity, mainly nephrotoxicity, has been shown and confirmed in published trials. It will be important to use this new cisplatin formulation in future trials and to test it in malignancies such as ovarian and bladder cancers. 

## Figures and Tables

**Table 1:** Pharmacokinetic parameters of total platinum in patients' sera at the different dose levels.

Dose	Pts	AUC_0*∅*A_	*C*_max _	Cl	*K*_el_	*t*_1/2_	*V*_*ss*_
mg/m^2^	(*n*)	(h *μ*g/mL)	(*μ*g/mL)	(L/m^2^ h)	(L/h)	(h)	(L/m^2^)
25	5	139.63	2.48 ± 1.18	0.18	0.0114	60.79	15.71
50	3	119.19	2.87 ± 0.59	0.42	0.0001	N/A	N/A
100	5	172.89	3.74 ± 1.18	0.58	0.0059	117.46	98.03
125	4	256.09	5.65 ± 2.67	0.49	0.0085	81.53	57.42

N/A, not applicable.

**Table 2:** Toxicity: lipoplatin monotherapy.

Dosage lipoplatin mg/m^2^	Toxicity	Grade			
		1	2	3	4
		*n*	*n*	*n*	*n*
150–250	Nausea-vomiting	—	—	—	—
	Fatigue	—	—	—	—
	Diarrhea	—	—	—	—
	Nephrotoxicity	—	—	—	—
	Neutropenia	—	—	—	—
	Neurotoxicity	—	—	—	—

300	Nausea-vomiting	2/4	1/4	—	—
	Fatigue	2/4	1/4	—	—
	Neutropenia	1/4	—	—	—
	Nephrotoxicity	1/4	—	—	—

350	Nausea-vomiting	1/4	3/4	—	—
	Fatigue	1/4	3/4	—	—
	Neutropenia	2/4	1/4	1/4	—
	Nephrotoxicity	2/4	1/4	1/4	—

**Table 3:** Toxicity/statistical differences.

Toxicity grade 1–4	Arm A	Arm B	
	*n *(%)	*n* (%)	*P* value*
Anemia	50 (43.9)	62 (54.9)	0.112
Leucopenia (neutropenia)	38 (33.3)	52 (45.2)	0.017
Thrombocytopenia	2 (1.8)	3 (2.6)	1.000^†^
Nephrotoxicity (renal)	7 (6.1)	46 (40.0)	<0.001
Neurotoxicity	52 (45.6)	63 (54.8)	0.145
GI toxic nausea/vomiting	37 (32.5)	52 (45.2)	0.042
GI diarrhea	2 (1.8)	3 (2.6)	1.000^†^
Asthenia	65 (57.0)	82 (71.3)	0.019
Alopecia	96 (84.2)	87 (75.7)	0.134

GI, gastrointestinal.

*Pearson's chi-square test.

^†^Fisher's exact test.

**Table 4:** Response rate/survival time (months), Log-rank test *P* value: 0.577.

		ARM			
Response rate		A	B	Total	*P* value*
CR					
*n *		1	0	1	—
% within ARM		0.9	0.0	0.4	—
PR					
*n *		67	54	121	
% within ARM		58.8	47.0	52.8	0.073
SD					
*n *		42	50	92	
% within ARM		36.8	43.5	40.2	0.306
PD					
*n *		4	11	15	
% within ARM		3.5	9.6	6.6	0.064

Total	*n*	114	115	229	

Survival time	*n*	Median		95% CI	

Arm A	114	9.0		6.2–11.8	
Arm B	115	10.0		6.8–13.2	

Total sample	229	10.0		8.3–11.7	

CR: complete response; PR: partial response; SD: stable disease; PD: progressive disease.

*Pearson's chi-square test.

**Table 5:** Response rate.

	Arm A	Arm B	*P* value
	(*n* = 103)	(*n* = 99)	
Partial response	61 (59.22%)	42 (42.42%)	0.036
Stable disease	35 (33.98%)	43 (43.43%)	0.220
Progressive disease	7 (6.80%)	14 (14.14%)	0.110

## References

[1] Einhorn LH, Williams SD, Loehrer PJ (1989). Evaluation of optimal duration of chemotherapy in favorable-prognosis disseminated germ cell tumors: a Southeastern Cancer Study Group protocol. *Journal of Clinical Oncology*.

[2] Aabo K, Adams M, Adnitt P (1998). Chemotherapy in advanced ovarian cancer: four systematic meta-analyses of individual patient data from 37 randomized trials. *British Journal of Cancer*.

[3] Pignon JP, Bourhis J, Domenge C, Designe L (2000). Chemotherapy added to locoregional treatment for head and neck squamous-cell carcinoma: three meta-analyses of updated individual data. *The Lancet*.

[4] Non-small Cell Lung Cancer Collaborative Group (1995). Chemotherapy in non-small cell lung cancer, a meta-analysis using updated data on individual patients from 52 randomized clinical trials. *British Medical Journal*.

[5] Kaufman D, Raghavan D, Carducci M (2000). Phase II trial of gemcitabine plus cisplatin in patients with metastatic urothelial cancer. *Journal of Clinical Oncology*.

[6] Rosenberg B, Lippert B (1999). Platinum complexes for the treatment of cancer: why the research goes on. *Cisplatin: Chemistry of a Leading Anticancer Drug*.

[7] Hayes DM, Cvitkovic E, Golbey RB, Scheiner E, Helson L, Krakoff  IH (1977). High dose cis-platinum diamminedichloride: amelioration of renal toxicity by mannitol diuresis. *Cancer*.

[8] Sorenson CM, Eastman A (1988). Mechanism of cis-diamminedichloroplatinum(II)-induced cytotoxicity: role of G2 arrest and DNA double-strand breaks. *Cancer Research*.

[9] Gandara DR, Nahhas NA, Adelson MD (1995). Randomized placebo-controlled multicenter evaluation of diethyldithiocarbamate for chemoprotection against cisplatinum-induced toxicities. *Journal of Clinical Oncology*.

[10] Arany I, Safirstein RL (2003). Cisplatin nephrotoxicity. *Seminars in Nephrology*.

[11] Taylor AE, Wiltshaw E, Gore ME, Fryatt I, Fisher C (1994). Long-term follow-up of the first randomized study of cisplatin versus carboplatin for advanced epithelial ovarian cancer. *Journal of Clinical Oncology*.

[12] Tognoni A, Pensa F, Vaira F (2002). A dose finding study of carboplatin and gemcitabine in advanced non-small cell lung cancer. *Journal of Chemotherapy*.

[13] Johnson BE (2000). Integration of new agents into the treatment of advanced non-small cell lung cancer. *American Society of Clinical Oncology Educational Book*.

[14] Shepherd FA, Dancey J, Arnold A (2001). Phase II study of pemetrexed disodium, a multitargeted antifolate and cisplatin as first-line therapy in patients with advanced non-small cell lung cancer. *Cancer*.

[15] Barlesi F, Pujal JL (2005). Combination of chemotherapy without platinum compounds in the treatment of advanced non-small cell lung cancer: a systemic review of phase III trials. *Lung Cancer*.

[16] Stathopoulos GP, Veslemes M, Georgatou N (2003). Paclitaxel and vinorelbine combination in advanced inoperable adenocarcinoma of the lung: a phase II study. *Anticancer Research*.

[17] Stathopoulos GP, Dimitroulis J, Antoniou D (2005). Front-line paclitaxel and irinotecan combination chemotherapy in advanced non-small-cell lung cancer: a phase I-II trial. *British Journal of Cancer*.

[18] Ardizzoni A, Boni L, Tiseo M (2007). Cisplatin- versus carboplatin-based chemotherapy in first-line treatment of advanced non-small-cell lung cancer: an individual patient data meta-analysis. *Journal of the National Cancer Institute*.

[19] Boulikas T, Stathopoulos GP, Volakakis N, Vougiouka M (2005). Systemic lipoplatin infusion results in preferential tumor uptake in human studies. *Anticancer Research*.

[20] Teni Boulikas Lipoplatin: a successful cisplatin formulation.

[21] Kuo MT, Chen HHW, Song IS, Savaraj N, Ishikawa T (2007). The roles of copper transporters in cisplatin resistance. *Cancer and Metastasis Reviews*.

[22] Huang G, Mills L, Worth LL (2007). Expression of human glutathione S-transferase P1 mediates the chemosensitivity of osteosarcoma cells. *Molecular Cancer Therapeutics*.

[23] Wei Q, Dong G, Franklin J, Dong Z (2007). The pathological role of Bax in cisplatin nephrotoxicity. *Kidney International*.

[24] Zhu J, Yang Y, Wu J (2007). Bcl-2 cleavages at two adjacent sites by different caspases promote cisplatin-induced apoptosis. *Cell Research*.

[25] Boulikas T, Vougiouka M (2003). Cisplatin and platinum drugs at the molecular level. (Review). *Oncology Reports*.

[26] Wang D, Lippard SJ (2005). Cellular processing of platinum anticancer drugs. *Nature Reviews Drug Discovery*.

[27] Villedieu M, Briand M, Duval M, Heron JF, Gauduchon P, Poulain L (2007). Anticancer and chemosensitizing effects of 2,3-DCPE in ovarian carcinoma cell lines: link with ERK activation and modulation of p21(WAF1/CIP1), Bcl-2 and Bcl-x(L) expression. *Gynecologic Oncology*.

[28] Singh M, Sharma H, Singh N (2007). Hydrogen peroxide induces apoptosis in HeLa cells through mitochondrial pathway. *Mitochondrion*.

[29] Berndtsson M, Hägg M, Panaretakis T, Havelka AM, Shoshan MC, Linder S (2007). Acute apoptosis by cisplatin requires induction of reactive oxygen species but is not associated with damage to nuclear DNA. *International Journal of Cancer*.

[30] Ohta T, Ohmichi M, Hayasaka T (2006). Inhibition of phosphatidylinositol 3-kinase increases efficacy of cisplatin in in vivo ovarian cancer models. *Endocrinology*.

[31] Fedier A, Erdmann R, Boulikas T, Fink D (2006). Potential of the Akt inhibitor LY294005 to antagonize the efficacy of cisplatin against HCT116 tumor cells in a DNA mismatch repair-dependent manner. *International Journal of Oncology*.

[32] Bodo J, Hunakova L, Kvasnicka P (2006). Sensitisation for cisplatin-induced apoptosis by isothiocyanate E-4IB leads to signalling pathways alterations. *British Journal of Cancer*.

[33] Stoeck A, Gast D, Sanderson MP, Issa Y, Gutwein P, Altevogt P (2007). L1-CAM in a membrane-bound or soluble form augments protection from apoptosis in ovarian carcinoma cells. *Gynecologic Oncology*.

[34] Arienti C, Tesei A, Ravaioli A (2008). Activity of lipoplatin in tumor and in normal cells in vitro. *Anticancer Drugs*.

[35] Fedier A, Poyet C, Perucchini D, Boulikas T, Fink D (2006). MLH1-deficient tumor cells are resistant to lipoplatin, but retain sensitivity to lipoxal. *Anticancer Drugs*.

[36] Devarajan P, Tarabishi R, Mishra J (2004). Low renal toxicity of lipoplatin compared to cisplatin in animals. *Anticancer Research*.

[37] Marr AK, Kurzman ID, Vail DM (2004). Preclinical evaluation of a liposome-encapsulated formulation of cisplatin in clinically normal dogs. *American Journal of Veterinary Research*.

[38] Froudarakis ME, Greillier L, Monjanel-Mouterde S (2010). Intrapleural administration of lipoplatin in an animal model. *Lung Cancer*.

[39] Tippayamontri T, Kotb R, Paquette B, Sanche L Cellular uptake and cytoplasm/DNA distribution of cisplatin and oxaliplatin and their liposomal formulation in human colorectal cancer cell HCT116.

[40] Charest G, Paquette B, Fortin D, Mathieu D, Sanche L (2010). Concomitant treatment of F98 glioma cells with new liposomal platinum compounds and ionizing radiation. *Journal of Neuro-Oncology*.

[41] Stathopoulos GP, Boulikas T, Vougiouka M (2005). Pharmacokinetics and adverse reactions of a new liposomal cisplatin (lipoplatin): phase I study. *Oncology Reports*.

[42] Stathopoulos GP, Boulikas T, Vougiouka M, Rigatos SK, Stathopoulos JG (2006). Liposomal cisplatin combined with gemcitabine in pretreated advanced pancreatic cancer patients: a phase I-II study. *Oncology Reports*.

[43] Froudarakis ME, Pataka A, Pappas P (2008). Phase 1 trial of lipoplatin and gemcitabine as a second-line chemotherapy in patients with nonsmall cell lung carcinoma. *Cancer*.

[44] Stathopoulos GP, Rigatos SK, Stathopoulos J (2010). Liposomal cisplatin dose escalation for determining the maximum tolerated dose and dose-limiting toxicity: a phase I study. *Anticancer Research*.

[45] Koukourakis MI, Giatromanolaki A, Pitakoudis M (2009). Concurrent liposomal cisplatin (lipoplatin), 5-fluorouracil and radiotherapy for the treatment of locally advanced gastric cancer: a phase I-II study. *International Journal of Radiation Oncology Biology Physics*.

[46] Karpathiou G, Argiana E, Koutsopoulos A, Froudarakis ME (2008). Response of a patient with pleural and peritoneal mesothelioma after second-line chemotherapy with lipoplatin and gemcitabine. *Oncology*.

[47] Jehn CF, Boulikas T, Kourvetaris A, Possinger K, Luftner D (2007). Pharmacokinetics of liposomal cisplatin (lipoplatin) in combination with 5-FU in patients with advanced head and neck cancer: first results of a phase III study. *Anticancer Research*.

[48] Farhat FS, Ibrahim K, Kattan J Preliminary results of phase II study of liposomal cisplatin—vinorelbine combination as first-line treatment in HER2/neu negative metastatic breast cancer (MBC).

[49] Mylonakis N, Athanasiou A, Ziras N (2010). Phase II study of liposomal cisplatin (lipoplatin) plus gemcitabine versus cisplatin plus gemcitabine as first line treatment in inoperable (stage IIIB/IV) non-small cell lung cancer. *Lung Cancer*.

[50] Stathopoulos GP, Antoniou D, Dimitroulis J (2010). Liposomal cisplatin combined with paclitaxel versus cisplatin and paclitaxel in non-small-cell lung cancer: a randomized phase III multicenter trial. *Annals of Oncology*.

[51] Stathopoulos GP, Antoniou D, Dimitroulis J, Stathopoulos J, Marosis K, Michalopoulou P Comparison of liposomal cisplatin versus cisplatin in non-squamous cell non-small cell lung cancer.

